# Depression in Dercum’s disease and in obesity: A case control study

**DOI:** 10.1186/1471-244X-12-74

**Published:** 2012-07-03

**Authors:** Emma Hansson, Henry Svensson, Håkan Brorson

**Affiliations:** 1 Department of Clinical Sciences Malmö, Lund University, Plastic and Reconstructive Surgery, Skåne University Hospital, SE-205 02, Malmö, Sweden

**Keywords:** Dercum’s disease, Adiposis dolorosa, Chronic pain, Obesity, Depression, MADRS

## Abstract

**Background:**

Dercum’s disease is characterised by pronounced pain in the adipose tissue and a number of associated symptoms. The condition is usually accompanied by generalised weight gain. Many of the associated symptoms could also be signs of depression. Depression in Dercum’s disease has been reported in case reports but has never been studied using an evidence-based methodology. The aim of this study was to examine the presence of depression in patients with Dercum’s disease compared to obese controls that do not experience any pain.

**Methods:**

A total of 111 women fulfilling the clinical criteria of Dercum’s disease were included. As controls, 40 obese healthy women were recruited. To measure depression, the Montgomery Åsberg Depression Rating Scale (MADRS) was used.

**Results:**

According to the total MADRS score, less than half of the patients were classified as having “no depression” (44%), the majority had “light” or “moderate depression” (55%) and one individual had “severe depression” in the Dercum group. In the control groups, the majority of the patients were classified as having “no depression” (85%) and a small number had “light depression” (15%). There was a statistically significant difference for the total MADRS score between the two groups (*p* = 0.014).

**Conclusion:**

The results indicate that the patients with Dercum’s disease are more likely to suffer from depression than controls.

## Background

Dercum’s disease is characterised by pronounced pain in the adipose tissue and a number of associated symptoms. The condition is usually accompanied by generalised weight gain. The pain is chronic (>3 months), symmetrical, often disabling and therapy-resistant to analgesics [1]. The pathogenesis of Dercum’s disease is unknown [1]. In 1901, Roux and Vitaut [2] proposed four cardinal symptoms of Dercum’s disease: (1) Multiple, painful, fatty masses (2) Generalised obesity (3) Weakness and susceptibility to fatigue (asthenia) (4) Psychiatric manifestations, including emotional instability, depression, epilepsy, confusion and dementia. However, it is still unclear which symptoms are cardinal and which are associated. In fact, already in 1927, Labbé and Boulin [3] questioned whether the weakness, susceptibility to fatigue, and psychiatric manifestations should be classified as cardinal symptoms. They argued that obesity *per se* can induce asthenia, and pointed out that psychiatric symptoms have not been described in all cases of Dercum’s disease [3].

In addition, patients with depression are often diagnosed with chronic pain conditions and vice versa [4]. Both disorders activate common neurocircuitries, such as the hypothalamic-pituitary-adrenal axis, limbic and paralimbic structures, ascending and descending pain pathways, and mutual neurotransmitters [5], and it is therefore sometimes difficult to determine whether the pain disorder or the psychiatric condition is the primary diagnosis. However, symptoms that could be attributed to depression have been described in patients in several reports to date [6].

The aim of the present study was to examine the presence of depression in Dercum’s disease compared to obese controls that do not experience any pain, thereby investigating whether depression is truly a part of Dercum’s disease. Depression was evaluated using The Montgomery Åsberg Depression Rating Scale (MADRS). It is thought that improving our knowledge about Dercum’s disease should improve patient care.

## Methods

### Patients and controls

A total of 111 patients fulfilling the clinical criteria of Dercum’s disease were diagnosed and referred to the Department of Plastic and Reconstructive Surgery in Malmö by the same consultant in internal medicine. Dercum’s disease was defined as adiposity and chronic pain (>3 months) in the adipose tissue [1]. Diagnosis was based on the medical history evaluated from a standardised questionnaire and a systematic physical examination on three separate visits. The purpose of the questionnaire and the examination was to exclude explanations other than Dercum’s disease, such as other pain conditions or endocrinological disorders, to the patients’ symptoms. All of the patients included in the study had generalised Dercum’s disease, meaning that they did not have any lipomas. The included subjects did not have any disease involving inflammation or pain, other than Dercum’s disease. The patients were referred to the Department of Plastic Surgery to be included in a study on the effect of liposuction on the pain experienced by the patients with Dercum’s disease. The first 53 consecutively-referred patients were later operated on with liposuction and the following 58 women with Dercum’s disease were recruited as controls to compare the effect of liposuction in the disease. The result of that study has been reported elsewhere [7]. All of the patients that were referred to this study were female. Healthy controls with a similar BMI, a similar age and the same sex were selected from patients with no acute or chronic pain, who were to undergo abdominoplasty surgery in the same department. In total, 40 women with a similar BMI and a similar age as the Dercum patients without any chronic diseases were recruited for this study. The patients’ profile is given in Table 1. During the first visit at the Department of Plastic and Reconstructive Surgery depression was evaluated in all of the patients and controls. None of the patients or controls had a diagnosis of depression and none of the patients or controls took antidepressants. None of the patients or controls had any disease that can give symptoms of depression.

**Table 1:** Patient profile (median, range)

**Baseline characteristics**	**Dercum patients (n = 111)**	**Control patients (n = 41)**
Age (years)	53 (22–73)	50 (26–69)
Weight (kg)	94 (55–147)	91 (55–129)
BMI (kg/m^2^)	34 (22–58)	34 (28–46)

### Ethics

The study was approved by the Ethics of Human Investigation Committee at Lund University (LU 236–89). All participants gave their written informed consent to participate. The procedures followed were in accordance with the Declaration of Helsinki of 1964, as revised.

### Montgomery Åsberg Depression Rating Scale (MADRS)

The MADRS is a self-rating scale that comprises nine different items, evaluating: (1) mood, (2) feelings of unease, (3) sleep, (4) appetite, (5) ability to concentrate, (6) initiative, (7) emotional involvement, (8) pessimism, and (9) zest for life. There is an apparent overlap between the MADRS items and the DSM-IV criteria for depression, although the MADRS does not directly evaluate the nine DSM-IV criteria. For instance, fatigue or loss of energy and psychomotor agitation or retardation are not directly assessed. The occurrence of suicidal thoughts was not included in the MADRS used in this study.

The use of items allows rating of individual symptoms and mood dimensions. Each item was given a score of 0–6, resulting in a total of 0–54 points. According to the interpretation guidelines, a total score of 0–12 points equalled “no depression”, 13–19 points implied a “light depression”, 20–34 points was “moderate depression”, and ≥35 points indicated “severe depression” [8].

A Swedish version of the MADRS has been validated and scores on the scale have been shown to correlate with scores on the Hamilton Rating Scale (HRS) [8] and the Beck Depression Inventory (BDI) [9]. The MADRS has previously been used to make the diagnosis of depression in patients with chronic pain conditions [10].

### Statistics

Histograms were drawn to examine the distribution of the measured factors. The histograms indicated that the measured factors were not normally distributed. Because of this, and the ordinal nature of the MADRS scale, values were given as median, ranges and percentages and the non-parametric Mann–Whitney *U*-test was used to compare MADRS scores between patients and controls.

## Results

**Figure 1:**
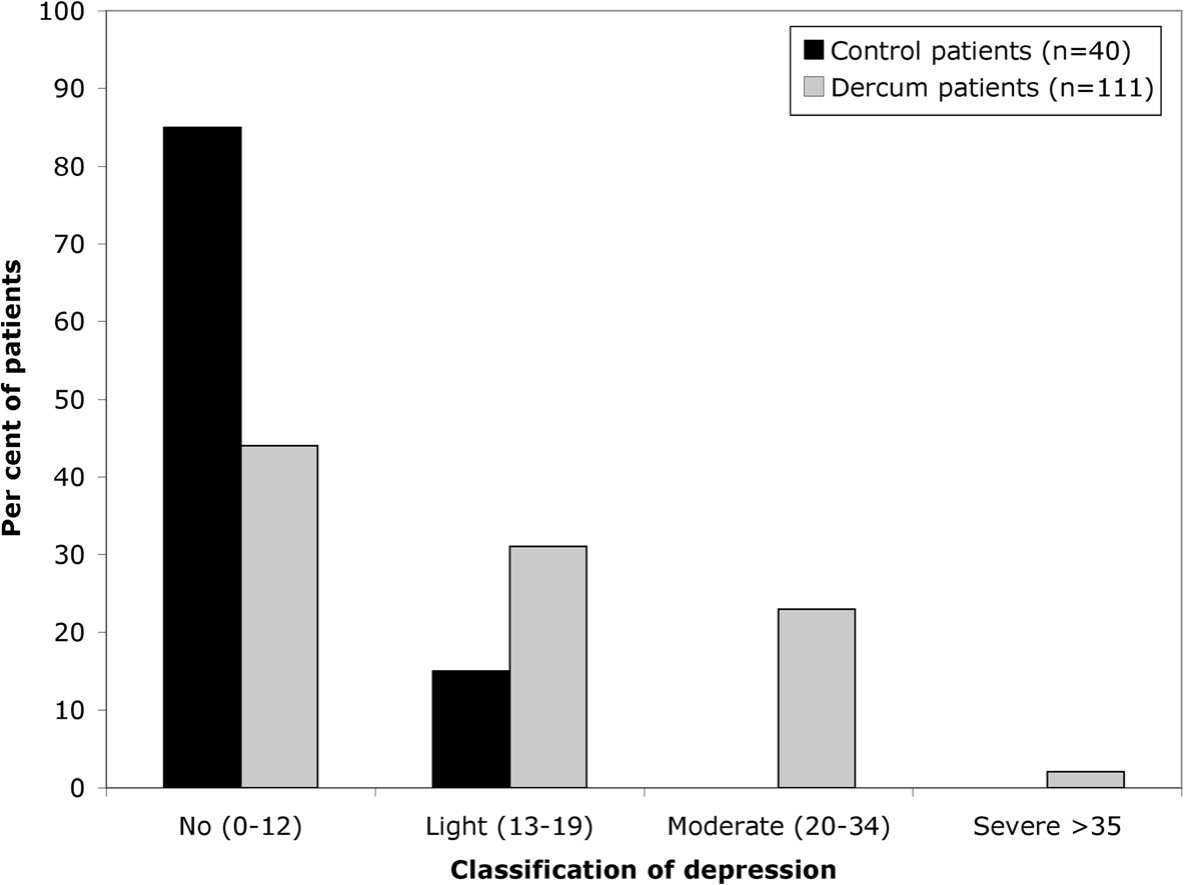
**Per cent of patients in the groups that received total scores in each interval.** According to the interpretation guidelines, a total score of 0–12 points equals “no depression”, 13–19 points a “light depression”, 20-34 points a “moderate depression”, and ≥35 points a “severe depression” [8]

According to the total MADRS scores, less than half of the patients were classified as having “no depression” (44%), the majority had light or moderate depression (55%), and one individual was suffering from “severe depression” in the Dercum group (Figure 1). In the control group, the majority of the patients were classified as having “no depression” (85%) and a small number had “light depression” (15%) (Figure 1). A statistical difference for the total score could be seen between the two groups (*p* = 0.014). For the items tested, a statistically significant difference was detected for “mood” (*p* = 0.018), “pessimism” (*p* = 0.022) and “zest for life” (*p* = 0.009). No statistically significant differences were seen for the rest of the items (Table 2).

**Table 2:** MADRS scores (median, range) and comparison between groups

**MADRS**	**Dercum**	**Control**	**Dercum patients vs. Control patients *p*-value**
	**patients (n = 111)**	**patients (n = 40)**	
Mood	1 (0–5)	0 (0–3)	0.018
Feelings of unease	2 (0–5)	0 (0–2)	0.40
Sleep	4 (0–6)	0 (0–4)	0.19
Appetite	0 (0–5)	0 (0–4)	0.60
Ability to concentrate	2 (0–5)	0 (0–2)	0.051
Initiative	2 (0–6)	0 (0–4)	0.092
Emotional involvement	2 (0–5)	0 (0–2)	0.074
Pessimism	1 (0–4)	0 (0–4)	0.022
Zest for life	1 (0–6)	0 (0–3)	0.009
Total score	14 (0–38)	4 (0–18)	0.014

## Discussion

Dercum’s disease is characterised by obesity, chronic pain and other associated symptoms. Some of the associated symptoms, previously described in case reports on Dercum’s disease [6], include depression and symptoms associated with depression, such as asthenia, weakness, fatigue, emotional instability, mental confusion, dementia, poor sleep quality and changes in appetite. Several studies have demonstrated that there is a significant association between depression and pain [5], as well as between depression and obesity [11]. Patients with depression are often diagnosed with chronic pain conditions and vice versa [5]. Some studies have demonstrated that depression predicts obesity later in life [11], and other studies support that obese subjects develop depression to a greater extent than subjects with lower, “normal” body weights [12]. Nonetheless, it is unclear whether there is a causal relationship between the three entities. The possible co-morbidity could be explained by *Berkson’s bias*[13], that is, patients with an illness might seek care more often. Thus co-morbidity could be overrepresented in a group of subjects that are, as in this study, recruited from a care setting. In summary, it is difficult to separate chronic pain from depression.

The elevated MADRS scores in the Dercum patients in this study cannot be explained by obesity alone, as the distribution of the Dercum subjects’ scores was different to that of the weight-matched control patients (Figure 1), and there was a statistical difference for the total score between the two groups (Table 2). The lack of statistical difference for a number of the items could be explained by low power, that is, by the small number of subjects in the control group (n = 40). The results suggested that the obese Dercum patients experience worse depression than obese healthy controls. As all of the Dercum patients had chronic pain whilst none of the controls had any history of chronic or present acute pain, it is unclear whether the depression is due to the Dercum’s disease *per se* or due to the experience of pain. Furthermore, it is unclear whether the depression or the Dercum’s disease came first. Previous research has shown that antidepressants have an effect on pain and the quality of life in patients with chronic pain [14] and that obese patients could benefit from the treatment of any co-existing features of depression [14,15].

An example of an associated symptom in Dercum’s disease that can also be explained by depression is poor sleep quality. Poor sleep quality can diminish an individual’s ability to cope with pain and stress and can influence the onset and course of disease [16]. In fact, a study on patients with chronic pain conditions demonstrated that sleeping less than 8 hours per 24 hours, especially in combination with poor sleep quality, might generate stronger reactions to pain [17]. In addition, Affleck et al. concluded that there is a correlation between sleep quality and experienced pain intensity, as well as the ability to cope with pain, among patients with fibromyalgia [18]. It can be speculated, therefore, that poor sleep can contribute to the onset of Dercum’s disease and the maintenance of pain. Conversely, obesity can also affect sleep quality [19]. Obstructive sleep apnoea (OSA) and Pickwick syndrome [1], both of which have been previously described in Dercum’s disease, can be explained by obesity, as 50% of otherwise healthy obese women with BMI >40 have OSA and more than 29% of severely obese patients have nocturnal hypoventilation [20]. This could explain why no difference can be seen between the Dercum patients and the control subjects in this study, as all of the subjects have similar BMIs.

One advantage of the present study is that the same consultant made the diagnosis of Dercum’s disease in all of the cases and that a control group of healthy obese subjects was included. Furthermore, an instrument was used to measure depression that has been previously validated and extensively used in research studies focusing on depression and patients with chronic pain [10]. A disadvantage is the fact that a normal weight control group was not included. However, there was an opportunity to include a control group of healthy obese subjects, which means that the hypothesis that signs of depression in Dercum’s disease could be explained by obesity alone can be excluded. Another weakness is the low number of patients included in this study, which is as a result of the rarity of the condition. Furthermore, it should be noted that the results are only valid for women as all of the subjects included were female.

## Conclusions

The relationship between Dercum’s disease, chronic pain, depression, and obesity is complex and it is not possible to separate depression and chronic pain completely. However, the results of this study indicate that patients with Dercum’s disease could suffer from worse depression than equally obese controls with no history of Dercum’s disease. This fact should be kept in mind when a treatment strategy for Dercum’s disease is selected.

## Competing interests

The authors declare that they have no competing interests.

## Authors’ contributions

EH participated in the design of the study, performed the statistical analysis and wrote the manuscript. HS participated in the choice of statistical methods and in the writing of the manuscript. HB initiated and designed the study and contributed to the writing of the manuscript. All authors have read and approved the final manuscript.

## Pre-publication history

The pre-publication history for this paper can be accessed here:

http://www.biomedcentral.com/1471-244X/12/74/prepub
